# Microbial content in Ayurvedic medicine

**DOI:** 10.4103/0974-7788.64397

**Published:** 2010

**Authors:** Reshmi Sarin

**Affiliations:** *Department of Dravyaguna, Arya Vaidya Chuikitsalayam and Research Institute, Coimbatore, India* E-mail: reshmi.pushpan@gmail.com

Sir,

This is with regard to the article, "Quality assessment of different marketed brands of *Dasamoolaristam*, an Ayurvedic formulation" by Kalaiselvan *et al*.[1]

The manuscript brings to our attention the very important issue of following stringent guidelines in the testing of Ayurveda medicines. However, there are some gross errors and misinformation in this article which need to be brought to the readers' attention.

In the introduction section, *Dasamoolarishtam*[2] is wrongly described as a formulation containing 10 herbs, which is incorrect. The original, traditional formulation contains 55 herbs as decoction, 11 herbs as powder, jaggery and honey.

It is important to note that Arya Vaidya Sala, Kotakkal and The Arya Vaidya Pharmacy Ltd., Coimbatore are two different, independent Ayurvedic institutions. The authors claim to have collected the samples from "Kotakkal Arya Vaidya Pharmacy, Coimbatore", which is a gross error in itself. First, it would be appropriate to provide the name of the actual institution accurately. Secondly, it is a known fact that neither of these two companies' outlets stock products of other brands, and hence it is impossible for the authors to have procured five different samples of *Dasamoolarishtam* from either of these two companies' outlets. Though these observations in no way alter their findings, the overall integrity of the study and thus the overall validity of the findings become questionable. The impact of such misreporting in a leading scientific journal like yours is a serious matter and could invite undeserved disrepute to these traditional houses of Ayurveda. These two institutions have been known for decades for their quality medicines and have a rich legacy of selfless service.

Another point is that the authors' statement on the alcohol content in the abstract and discussion sections contradict each other. The Ayurvedic Pharmacoepia of India, Part II, Volume II, says that the formulation "contains not more than 10 percent, and not less than 5 percent of alcohol that is self generated in the preparation over a period of time."
